# Neural circuits and emotional processing in rapid eye movement sleep

**DOI:** 10.3389/fpsyt.2025.1674071

**Published:** 2025-12-05

**Authors:** Liu-Yan Chang, Yue-Qian Wang, Zhe Li, Yang Zhang, Zhi-Li Huang, Su-Rong Yang

**Affiliations:** Department of Pharmacology, School of Basic Medical Sciences; Fudan University, Shanghai, China

**Keywords:** rapid eye movement sleep, neural circuit, anxiety, depression, fear

## Abstract

Mammalian sleep consists of non-rapid eye movement sleep (NREMS) and rapid eye movement sleep (REMS), accounting for approximately 75% and 25% of total sleep, respectively. REMS is characterized by low-amplitude and high-frequency theta oscillations in the brain, muscle atonia, intermittent muscle twitches, rapid eye movements, and rapid breathing. Although relative brief in duration, REMS is evolutionarily conserved across species. Notably, REMS plays a critical role in emotion regulation and its dysregulation has been closely associated with neuropsychiatric disorders such as post-traumatic stress disorder (PTSD) and depression. However, the precise neural mechanisms that initiate and terminate REMS, as well as the exact pathophysiological relationships between REMS and psychiatric conditions, remain poorly understood. In recent years, research on the circuitry and functional roles of REMS has advanced considerably, with growing evidence implicating several cortical and brainstem regions in its regulation. Here, we review the mechanisms of mammalian REMS in terms of brain anatomy and neural circuits, which constitute highly distributed networks spanning the cortex, brain stem, hypothalamus, and other regions. We also summarize the role of REMS in negative emotion processing. Finally, we propose key open questions that need to be addressed in future studies.

## Introduction

Humans spend one-third of their lives in sleep, which is crucial for maintaining normal physiological functions. There are two states of human sleep, non-rapid eye movement sleep (NREMS) and rapid eye movement sleep (REMS), distinguishable through electroencephalogram (EEG), electromyogram (EMG), and eye movement tracking. REMS was discovered and defined in 1953 by recording electrooculograms and observing periodic rapid eye movements and twitching during sleep in humans (1). Further, a significant correlation between these peculiar eye movements and dreaming was discovered (1). REMS, though it accounts for about one-fourth of human sleep time, has unique physiological significance and is indispensable for emotion processes in humans (2, 3). Clinically, REMS disorders may lead to conditions such as rapid eye movement sleep behavior disorder (RBD) and narcolepsy, but the pathogenesis remains unclear. Therefore, researching the neural mechanisms of REMS can provide a theoretical basis for precise clinical treatment of related disorders. In recent years, rapid developments in chemogenetics, optogenetics, and calcium imaging have enabled high-resolution fine-tuning of sleep-related neurons. Researchers using these novel techniques have identified additional brain regions and circuits that regulate REMS, leading to rapid progress in the study of REMS. This review is based on a comprehensive literature search conducted in major academic databases, including PubMed and Web of Science, for articles published between 2010 and 2025. Following a rigorous assessment of the quality and relevance of the retrieved full-text articles, we synthesize key recent research advances. Our analysis focuses on the neural regulatory mechanisms of REMS and its impact on emotions. We also propose unresolved critical questions. This article will provide a theoretical basis for exploring new strategies in the treatment of REMS disorders.

## Neural circuits for REMS

### Cortical regulation of REMS

Classical studies in cats by Michel Jouvet and others demonstrated that the brainstem is necessary and sufficient for REMS generation, as mesopontine lesions abolished REMS while cortical lesions or transections above the brainstem preserved NREMS-REMS cycles (4). However, recent studies have revealed complementary roles of the cortex in REMS regulation (5). For instance, Wang and colleagues employed mesoscale calcium imaging and optogenetics in mice to demonstrate for the first time the essential role of the occipital cortex in promoting the transitions from NREMS to REMS and in REMS homeostasis—a phenomenon wherein selective deprivation of REMS leads to an elevated REMS pressure and a subsequent increase in REMS amount during recovery sleep (6). In mice, calcium imaging showed heightened activity in the occipital cortex during a pattern designated as the “REMS-like state”. In contrast, the state characterized by increased neuronal activity in other brain regions, with the exception of the occipital cortex, is referred to as the “REMS-opponent state” (7). Oscillations between REMS-like and REMS-opponent state were observed in cortical activity during NREMS, indicating the presence of REMS pressure and suggesting the role of the occipital cortex in the homeostatic regulation of REMS. Optogenetic inhibition of occipital cortical activity reduced the transitions from NREMS to REMS and significantly shortened REMS duration. Conversely, activation of occipital cortical activity produced opposite effects, indicating its bidirectional role in regulating REMS-NREMS transitions (7).

The occipital cortex includes the retrosplenial cortex (RSC) and the visual cortex. Evidence suggests that the RSC is notably active during REMS, with increased neuronal firing rates (8–10). Using TRAP2 (targeted recombination in active populations) mice to label neurons active during REMS and wakefulness, it was found that the active neurons during REMS are primarily located in the superficial layers of the RSC (11). Another study found more precise regulation of REMS by the RSC during its two sub-stages of REMS: quiescent REMS (qREMS), characterized by low motor activity and slower theta oscillations (6.5–7.5 Hz), and active REMS (aREMS), which exhibits prominent phasic activities such as eye movements, facial twitches, and whisker movements, along with faster theta oscillations (8.2–10 Hz) (12). Dong et al. used large-field calcium imaging and two-photon imaging to find that the RSC is the starting point of cortical calcium activity waves during REMS, with selective activation of pyramidal neurons in layers 2/3 of the RSC during REMS (12). However, RSC neuronal activity patterns differ during REMS, activating at different stages, matching the transition from qREMS to aREMS. Optogenetic inhibition of the excitatory neurons in the RSC could suppress the transition between REMS sub-stages, shortening REMS duration, whereas inhibition during NREMS did not induce a sleep phase transition. These results reveal the role of the RSC in maintaining REMS and in the transition between its sub-stages (12). Retrograde tracing in the RSC revealed projections from the lateral pontine area, a key site for REMS generation, suggesting a connection between the RSC and the pontine area in REMS regulation. The RSC also receives projections from the hippocampus and may be involved in the generation of hippocampal theta oscillations (9). The RSC has connections to the claustrum (CLA) and the medial septum (MS), which may participate in memory consolidation and dream occurrence (5, 11).

The aREMS state reported by Dong et al. features enhanced theta-phase coupling of fast oscillations and eye movements (12), consistent with findings from other mouse and rat REMS sub-stages studies (13, 14). However, it is important to note that the classification of REMS sub-stages differs between mice and humans. In humans, classification is based on the presence of rapid eye movements during REMS; periods with eye movements are defined as phasic REMS, while periods without are defined as tonic REMS (15, 16). These sub-stages also have distinct physiological functions. For example, respiratory rate is slower during tonic REMS in humans (17) and also during short REMS episodes in mice, when tonic REMS predominate (18). Additionally, phasic REMS is associated with a reduction in environmental awareness (16), and both tonic and phasic REMS are implicated in disorders such as RBD and epilepsy (19–22). More specifically, in patients with RBD, violent dream-enactment behaviors frequently occur during phasic REMS, which coincides with aberrant activation of the motor cortex (19, 20). Furthermore, during phasic REMS, there is a marked suppression of the generation and propagation of interictal spikes and pathological high-frequency oscillations, contributing to the suppression of epileptic activity (21, 22). Therefore, investigating REMS sub-stages is crucial for elucidating the physiological functions of REMS and advancing therapeutic strategies for related disorders.

Recent studies indicate that excitatory pyramidal neurons in the medial prefrontal cortex (mPFC) become more active during the initiation and maintenance of REMS. Optogenetic activation of these neurons in mice facilitates the transition from NREMS to REMS and prolongs REMS duration via projections to the lateral hypothalamus (LH), possibly by engaging local melanin-concentrating hormone (MCH) neurons which are known REMS-promoting population (23, 24). In contrast, activation of inhibitory neurons in the mPFC reduces theta oscillations and shortens REMS, promoting transitions from REMS to wakefulness (23), possibly through suppression of REMS-active inhibitory neurons in the posterior LH (25). However, the specific subpopulations in the LH that mediate the opposing effects of mPFC excitatory and inhibitory neurons on REMS remain unidentified, warranting further investigation. Additionally, cFos expression in the anterior cingulate cortex (ACC), medial entorhinal cortex, and the hippocampal dentate gyrus (DG) in mice increased during REMS hypersomnia following REMS deprivation (REMSD). The activation of these cortical neurons is due to inputs from glutamate neurons of the CLA, GABA/glutamate neurons of the supramammillary nucleus (SuM), and GABAergic neurons of the MS (5, 11, 26).

In summary, certain cortical areas are activated during REMS. They participate in the initiation and regulation of REMS by functioning through local cortical microcircuits or subcortical deep nuclei. Among them, the RSC potentially receives direct inputs from the pons and relays signals to other cortical areas like primary visual cortex and ACC. Collectively, they govern both the transition from NREMS to REMS and the progression between different REMS substages. However, a deeper understanding of the cortical mechanisms underlying REMS regulation and the generation of distinct theta oscillation patterns is needed.

### Brainstem regulation of REMS

The brainstem, a key site for subcortical regulation of REMS, is composed of the midbrain, pons, and medulla oblongata, containing several nuclei involved in the generation, maintenance, and regulation of REMS. The laterodorsal tegmental nucleus (LDT) and pedunculopontine tegmental nucleus (PPT) within the pons are identified as areas of REMS initiation, comprising cholinergic, glutamatergic, and GABAergic neurons (27–29). The cholinergic neurons of LDT/PPT are recognized as REMS-ON neurons. Optogenetic activation of these neurons in mice can induce transitions from NREMS to REMS, increasing the frequency of REMS episodes (30). Advances in calcium imaging have revealed active glutamatergic neurons in the LDT during REMS (31). Chemogenetic activation of GABAergic PPT neurons slightly reduced REMS (29). Recent research indicates that signals from substantia nigra dopaminergic neurons and PPT cholinergic neurons may integrate into the amygdala to increase REMS (32). The sublaterodorsal nucleus (SLD), located ventrally to the aqueduct and periaqueductal gray and also known as the subcoeruleus, plays a crucial role in generating REMS and its characteristic atonia—the temporary paralysis of skeletal muscle (33, 34). The neurons in the SLD are active during REMS and immunohistochemical staining of rat brain slices after REMSD reveals that most cFos+ neurons in the SLD are glutamatergic (35). The SLD receives projections from LDT/PPT cholinergic neurons (36) and LH orexin neurons, thus enhancing its output and gap junction conductance and consolidating the brain’s active state (37). The SLD projects to the medulla, potentially linked to atonia production during REMS. The latest research shows that ablation of neurons expressing corticotropin-releasing hormone-binding protein (Crhbp) in the SLD specifically reduces REMS and impairs muscle atonia during REMS in mice (38). Crhbp+ neurons in the SLD that project to the medulla promote REMS. Within the medullary area receiving projections from Crhbp+ neurons, neurons expressing nitric oxide synthase 1 (Nos1) project to the SLD and promote REMS, suggesting a positively interacting loop between the pons and the medulla operating as a core REMS circuit (38).

The medulla oblongata is another important brain region involved in REMS regulation. It has been shown that glycinergic/GABAergic neurons in the ventral medulla (VM) hyperpolarize somatic motoneurons in the spinal cord and induce muscle atonia during REMS. In mice, rats, and cats, inactivation of these inhibitory neurons in the ventromedial medulla (vmM) induced abnormal motor behaviors during REMS, resembling RBD symptoms (34, 39, 40). VM GABAergic neurons in mice are active during REMS, relieving the ventrolateral periaqueductal gray (vlPAG) GABAergic inhibition on REMS-promoting nuclei and promoting REMS (41). GABAergic neurons in the dorsomedial medulla (dmM) of mice also promote the initiation and maintenance of REMS, likely through projections to the midline nuclei (42). The lateral and dorsal paragigantocellular nuclei (LPGi/DPGi) GABAergic neurons may receive projections from the SLD, inhibiting downstream REMS-off neurons like dorsal raphe nucleus (DRN) serotonergic and locus coeruleus (LC) noradrenergic neurons to promote REMS (43–46).

The rostromedial tegmental nucleus (RMTg), a midbrain region also known as the tail of the ventral tegmental area (VTA), is primarily composed of GABAergic neurons. Our previous work identified it as a crucial region for the initiation and maintenance of NREMS (47). Recent studies further reveal that RMTg GABAergic neurons play a significant role in terminating REMS. Optogenetic activation of these neurons during REMS promptly transitions mice to brief wakefulness; conversely, their inhibition converts NREMS into REMS and prolongs its duration (48). Electrophysiological recordings show that the activity of RMTg GABAergic neuron is low at REMS onset but increases with REMS duration, reflecting accumulation of REMS pressure. These neurons facilitate transitions from REMS to wakefulness by disinhibiting glutamatergic neurons in the LDT (48). The vlPAG GABAergic neurons in the midbrain also play significant roles in REMS and are predominantly REMS-off neurons. These neurons terminate REMS by inhibitory projections to downstream pontine REMS-on neurons. They receive upstream inhibitory projections from LH MCH neurons, VM GABAergic neurons, etc., thereby inhibiting REMS (41, 49, 50). A latest study demonstrates that activation of GABAergic neurons in the dorsal part of the deep mesencephalic reticular nucleus (dDpMe) rapidly terminates REMS, whereas their inhibition induces REMS. These neurons exert REMS by projections to the SLD and LH (51). Additionally, activating dDpMe GABAergic neurons prevents cataplexy in mice with damaged hypothalamic orexinergic neurons, offering significant insights into the pathophysiological mechanisms of cataplexy (51).

In short, the brainstem plays a critical role in the generation and maintenance of REMS, as well as in the regulation of muscle tone changes during REMS. An increasing number of brainstem nuclei have been identified as involved in its regulation. Future research is needed to further explore the heterogeneity of brainstem neurons.

### Hypothalamic regulation of REMS

Neurons in the LH critically regulate REMS initiation and maintenance. MCH neurons promote REMS by inhibiting REMS-off neurons in the vlPAG/dDpMe and wake-promoting histaminergic neurons in the tuberomammillary nucleus (TMN) (24, 52), while orexinergic neurons activate the SLD to maintain REMS and its homeostasis (37, 53). Located in the anterior hypothalamus, the preoptic area (POA) contains several subtypes of GABAergic neurons, including corticotropin-releasing hormone (CRH) and cholecystokinin neurons. These GABAergic neurons promote REMS by projections to the TMN (54). Lateral preoptic (LPO) neurons exhibit peak activity during REMS. Furthermore, mice with deleted GluN1 NMDA receptor subunit from LPO have strongly reduced cortical theta oscillations during REMS, suggesting that theses neurons are essential for generating REMS theta power (55). The dorsomedial hypothalamic nucleus contains galanin-positive GABAergic neurons that either promote REMS via projections to the raphe pallidus or inhibit it via projections to the POA (56). Located in the posterior hypothalamus, the lateral SuM projects to the DG, co-releasing GABA and glutamate (57, 58). Activation of the SuML-DG pathway in mice increases theta and gamma power during REMS (57). Additionally, LIM homeodomain factor (Lhx6)-expressing GABAergic neurons in the mouse zona incerta are highly active during REMS. These neurons bidirectionally regulate REMS, as ablation suppresses REMS, while optogenetic stimulation promotes it (59–61). Therefore, during REMS, the hypothalamus regulates REMS and its characteristic waveform changes through its interconnections and interactions with the brainstem and cortex.

### Other nuclei regulating REMS

Beyond the cortex, brainstem, and hypothalamus, REMS is modulated by other brain regions. In the olfactory bulb of rats, an adenosine A_2A_ receptor antagonist increases REMS, while an A_2A_ receptor agonist suppresses it (62). Similarly, infusion of a cannabinoid (CB1) receptor agonist into the MS of rats promotes REMS (63). In the basal lateral amygdala (BLA), a transient dopamine increase terminates NREMS and initiates REMS in mice by acting on BLA dopamine receptor D_2_ (Drd2)-expressing neurons (64). The entopeduncular nucleus (EP) contributes to REMS promotion in mice via somatostatin (Som) neurons that project to the lateral habenula (LHb) (65). Additionally, LHb glutamatergic neurons in mice are preferentially active during REMS. Their ablation induces a significant decrease in REMS, whereas their activation increases it (66). Although some studies report that CLA neurons are activated during REMS in rats (5, 26), *in vivo* two-photon calcium imaging reveals that those projecting to the RSC are suppressed during this state in mice (67).

**Figure 1:**
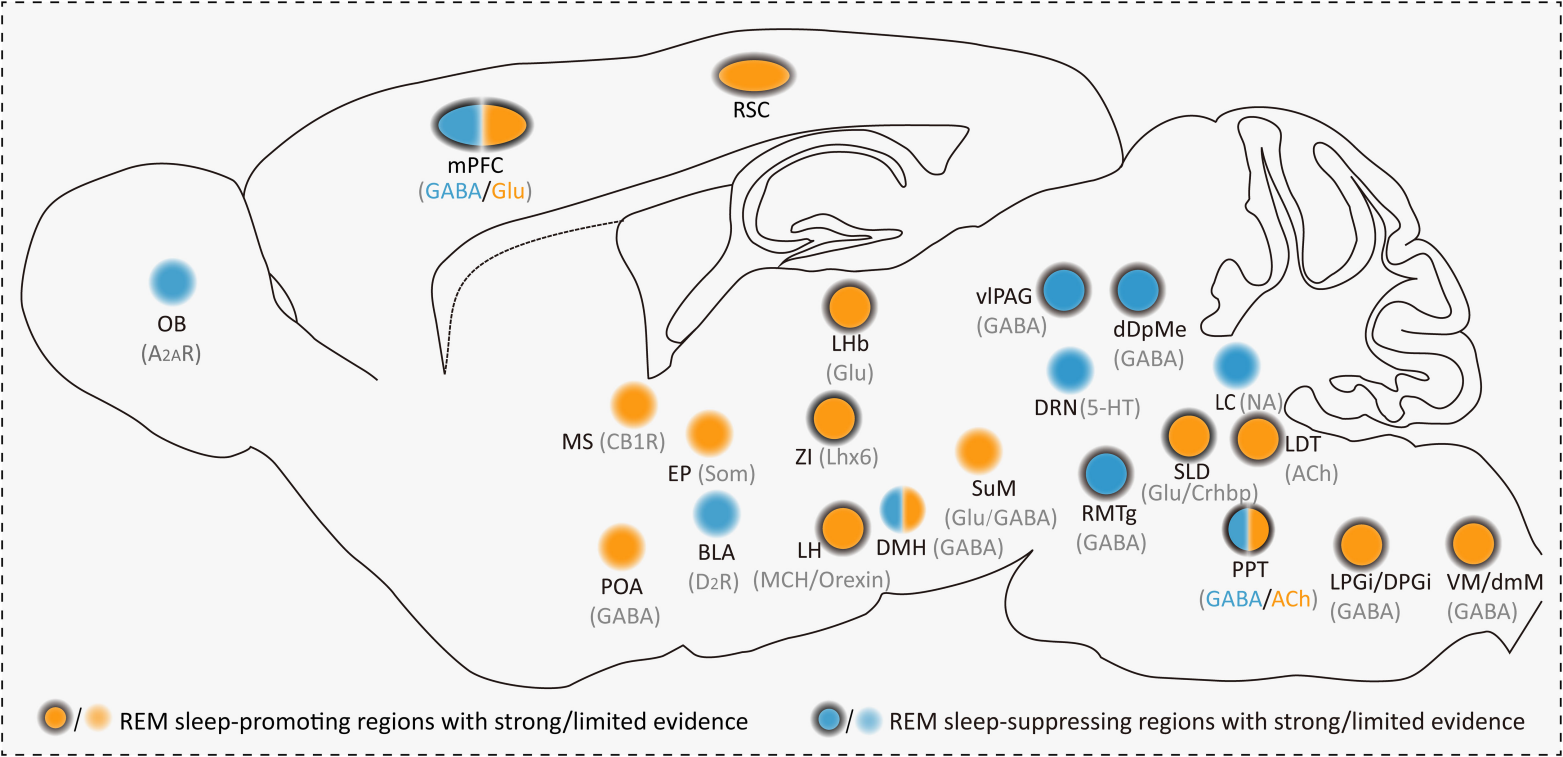
Summary of REMS-promoting/suppressing brain regions in rodent research. Neuronal types include the A_2A_R, CB1R, D_2_R, Som, GABA, MCH, Orexin, Lhx6, 5-HT, ACh, NA, Crhbp, GABA, and Glu. REMS-promoting brain regions with strong (orange with outer circle) and limited (orange without outer circle) evidence include the mPFC, RSC, MS, EP, LHb, POA, ZI, LH, DMH, SuM, SLD, LDT, PPT, LPGi, DPGi, VM, and dmM. REMS-suppressing brain regions with strong (blue with outer circle) and limited (blue without outer circle) evidence include the OB, mPFC, BLA, vlPAG, DRN, RMTg, DMH, dDpMe, LC, and PPT. “Strong evidence” implies the establishment of clear causal evidence in rodents and “Limited evidence” indicates that only correlational information exists, with causality yet to be determined. A_2A_R, adenosine 2A receptor-expressing; CB1R, cannabinoid receptor 1-expressing; D_2_R, dopamine receptor D_2_-expressing; Som, somatostatin; MCH, melanin-concentrating hormone-expressing; Orexin, orexinergic; Lhx6, the LIM homeodomain factor; 5-HT, serotonergic; ACh, cholinergic; NA, noradrenergic; Crhbp, corticotropin-releasing hormone-binding protein; GABA, GABAergic; Glu, glutamatergic. mPFC, medial prefrontal cortex; RSC, retrosplenial cortex; MS, medial septum; POA, preoptic area; EP, entopeduncular nucleus; LHb, lateral habenula; ZI, zona incerta; LH, lateral hypothalamus; DMH, dorsomedial hypothalamic nucleus; SuM, supramammillary nucleus; SLD, sublaterodorsal nucleus; LDT, laterodorsal tegmental nucleus; PPT, pedunculopontine tegmental; LPGi/DPGi, lateral and dorsal paragigantocellular nuclei; VM, ventral medulla; dmM, dorsomedial medulla; OB, olfactory bulb; BLA, basal lateral amygdala; vlPAG, ventrolateral periaqueductal gray; DRN, dorsal raphe nucleus; RMTg, rostromedial tegmental nucleus; dDpMe, deep mesencephalic nucleus; LC, locus coeruleus.

In all, the neural network underlying REMS regulation is summarized in **Figure 1**.

## REMS and emotion

Emerging evidence indicates that REMS plays a crucial role in emotion processing. Disruption of REMS is linked to affective disorders, including anxiety and depression (68–71). Conversely, emotional disorders can result in REMS abnormalities (72).

### REMS and anxiety

REMS influences anxiety through multiple mechanisms, including neurotransmitters, signaling pathways, and special neural circuits (73). For instance, chronic REMSD during adolescence elevates norepinephrine and serotonin levels in the amygdala and hippocampus, impairing physical development and inducing anxiety-like behaviors in rats (74). REMSD also induces anxiogenic effects by triggering neuroinflammatory responses, which involve the activation of signaling pathways such as NF-κB and tumor necrosis factor, along with microglial activation in the PFC (75). The link between REMS disruptions and anxiety is further supported by evidence from specific circuit manipulations. Local ablation of SLD neurons reduces REMS and increases anxiety-like behaviors (76). Chemogenetic inhibition of LHb glutamatergic neurons chronically inhibits REMS and also elevates anxiety in mice (65). It is critical to note that the stress inherent in REMSD methodologies confounds these interpretations (71). Future research requires low-stress models to clarify the specific role of REMS in anxiety pathogenesis.

### REMS and depression

Sleep disturbances, particularly REMS abnormalities such as shortened latency and prolonged duration, are common symptoms of depression (77–82). However, the precise mechanisms by which REMS affects depression remains unclear (83). Evidence suggests that endogenous substances and their signaling pathways may link REMS and depression. On one hand, acute REMSD in mice decreases brain-derived neurotrophic factor (BDNF) levels in serum and brain regions such as the PFC, PPT, and hippocampus in mice and induces depressive-like behaviors (84). Moreover, treatment with the antidepressant escitalopram elevates BDNF levels and ameliorates depressive-like behaviors in REMS-deprived mice (85). On the other hand, short-term REMSD can also have antidepressant effects in rats subjected to chronic unpredictable mild stress. This improvement is mediated by enhanced adenosine signaling and increased phosphorylation/expression of CREB1 (cyclic-AMP response element-binding protein 1)/YAP1 (Yes-associated protein 1)/c-Myc axis (81), or via inhibition of mPFC VIP neurons to increase pyramidal neuron excitability (86). The orexin system is also implicated in REMS abnormalities associated with depression (87). Administration of orexin A in the vmPFC exerts antidepressant effects, reversible by Orexin1R and TrkB receptor antagonists, suggesting orexin influences depressive behaviors via downstream TrkB-mediated signaling pathways (88). Furthermore, potentiation of neuronal activity in the LHb was associated with increased REMS in a depression mouse model induced by restraint stress (66).

Patients with depression frequently exhibit a shortened REMS latency and a prolonged REMS duration in clinical studies (78, 89, 90). Additionally, individuals with major depressive disorder (MDD) often show increased REM density—characterized by a greater frequency of rapid eye movements during REMS, particularly in the first REMS period (91–94). However, clinical observations have yielded divergent findings. For instance, Liu et al. reported that MDD patients exhibited extended REMS latency and reduced REMS duration (95), whereas Fuente et al. found no significant difference in REMS latency between MDD patients and healthy controls (96). These discrepancies may arise from variations in patient demographics, such as age, sex, and comorbidities. Emerging neuroimaging evidence illuminating the close relationship between MDD and REMS may offer a potential explanation for this phenotypic heterogeneity. Multimodal magnetic resonance imaging (MRI) analyses have linked cortical thinning in limbic regions with the severity of depressive symptoms (97). Furthermore, baseline REMS amount in MDD patients has been correlated with symptomatic improvement, potentially mediated through regulating neural activity in the left inferior temporal gyrus and cerebral blood flow in the bilateral paracentral lobule (83). Another MRI study associated higher REMS latency and less REMS amount with reduced voxel-mirrored homotopic connectivity in the precentral gyrus and inferior parietal lobule in MDD patients (95). These insights suggest that MRI analysis of the relationship between REMS abnormalities and brain functional connectivity in MDD patients can predict disease severity and prognosis. Moreover, clinically targeting these specific altered brain regions with neuromodulation to restore normal structure and function may circumvent the adverse effects of systemic pharmacotherapy.

### REMS and fear

REMS plays a critical role in fear memory processing, relying on brain activity in regions like the hippocampus, amygdala, and cortex. Its disruptions can dysregulate this process and lead to excessive fear memory consolidation and impaired extinction, ultimately contributing to the development of post-traumatic stress disorder (PTSD) (98–100). It is characterized by symptoms of traumatic re-experiencing, avoidance, and negative emotions, frequently develops in disaster survivors and military veterans as a consequence of major traumatic exposure (101–104). Clinically, PTSD patients frequently exhibit REMS disturbances, including increased frequency but shorter duration of REMS (105–107). By synthesizing evidence from both basic and clinical research on the relationship between REMS and fear memory, this review aims to provide insights that could inform therapeutic strategies for REMS-related fear disorders such as PTSD.

Basic research shows that compared to NREMS, mice awaken more readily to predator odor during REMS, indicating heightened responsiveness to threatening stimuli and environmental danger detection (108). This heightened arousal is mediated by the activation of CRH-positive neurons in the medial subthalamic nucleus, as chemogenetic or optogenetic inhibition of these neurons significantly prolongs awakening latency upon predator odor exposure in mice. Beyond immediate threat detection, REMS also plays a critical role in fear memory processes including the acquisition, consolidation, and extinction. Several brain regions such as the MS, SLD, and cortex are involved. Selective optogenetic silencing of GABAergic neurons in the MS during post-learning REMS impairs fear memory acquisition (109), while SLD lesions induced by diphtheria toxin A in rats or specific ablation of SLD glutamatergic neurons in mice completely abolish REMS and significantly enhance fear memory consolidation (110). Furthermore, the activity of pyramidal neurons in the infralimbic cortex during REMS promotes fear memory extinction, as inhibiting these neurons specifically during REMS disrupts this process (111).

Clinical evidence also supports the important role of REMS in fear memory processing. In one study, participants underwent fear acquisition tasks and were assigned to either sleep deprivation (SD) or normal sleep conditions. Post-awakening tests showed that SD impaired fear memory recall, and consolidation strength was positively correlated with REMS duration (112). Theta activity during REMS has been linked to hippocampal reactivation during this process (113), and computational modeling suggests that simulated theta oscillation may help to reduce forgetting (114). However, the role of REMS in fear consolidation remains controversial. Another study found that reactivation of conditioned stimuli during later REMS after fear acquisition does not affect fear memory consolidation (115). On the other hand, REMS facilitates fear extinction. When subjects underwent partial REMSD after fear acquisition, they showed impaired discrimination of threatening stimuli and weakened extinction memory (112). Neuroimaging studies suggest that REMS-dependent fear memory consolidation and extinction may involve hyperactivity in fear-related regions such as the basolateral amygdala and ACC, and extinction-related regions such as the prefrontal and infralimbic cortex, respectively (111, 112, 116). Although excessive fear generalization is a hallmark of PTSD, the role of REMS in this process requires further clarification. Some clinical evidence indicates that REMS can enhance discrimination between threat and safety signals, thereby inhibiting fear generalization (117).

Overall, REMS exerts multifaceted influences on fear processing. Further basic and clinical research is warranted to elucidate how REMS and its characteristic theta oscillations contribute to fear regulation.

**Figure 2:**
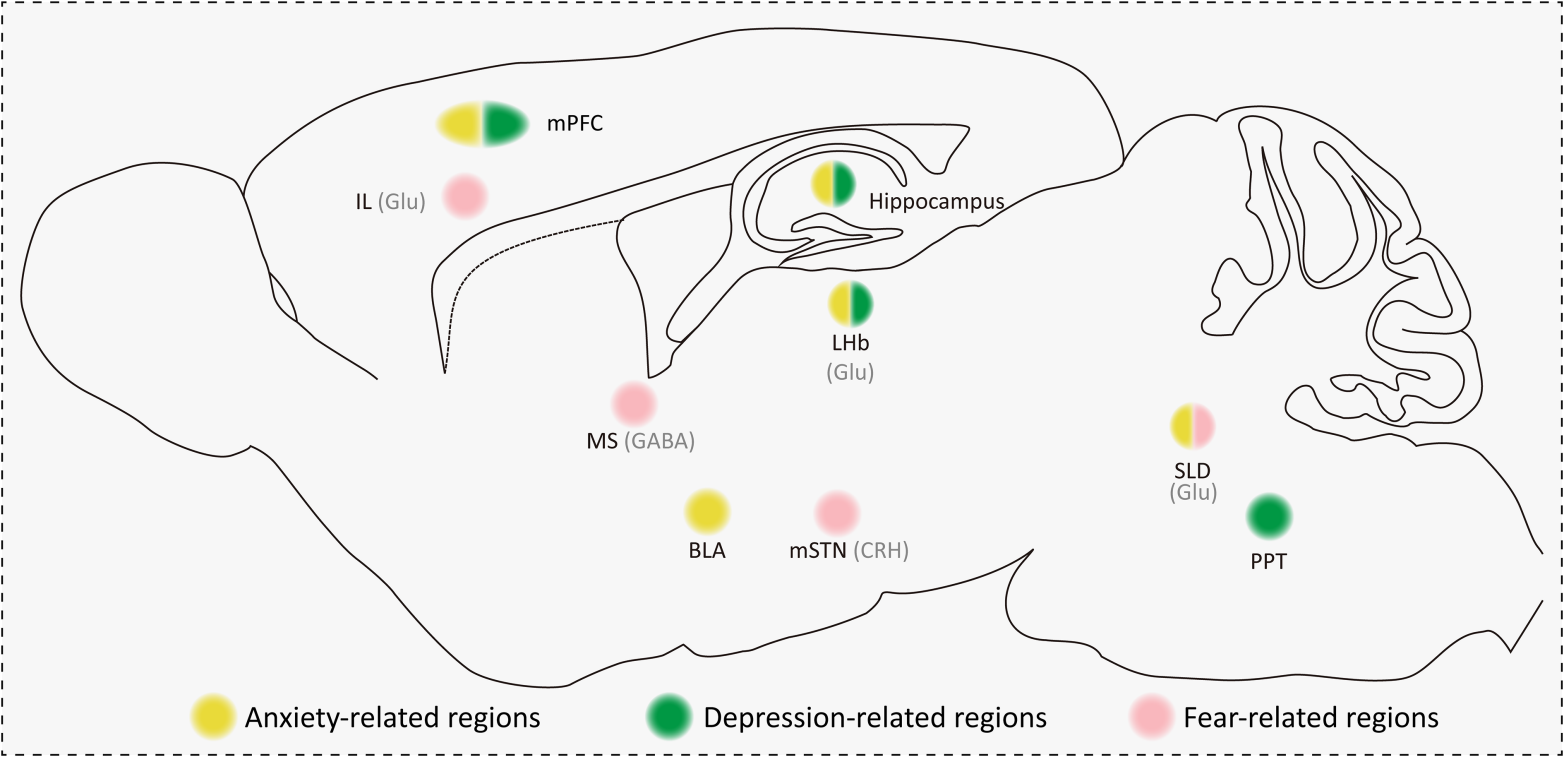
Summary of anxiety-, depression-, and fear-related brain regions in the context of aberrant REMS in rodent research. Neuronal types include Glu, GABA, and CRH. Anxiety-related brain regions (yellow circuits) include the mPFC, hippocampus, BLA, LHb, and SLD. Depression-related brain regions (green circuits) include the mPFC, hippocampus, LHb, and PPT. Fear-related brain regions (pink circuits) include the IL, MS, mSTN, and SLD. Glu, glutamatergic; GABA, GABAergic; CRH, corticotropin-releasing hormone. IL, infralimbic cortex; mPFC, medial prefrontal cortex; MS, medial septum; BLA, basal lateral amygdala; LHb, lateral habenula; mSTN, medial subthalamic nucleus; SLD, sublaterodorsal nucleus; PPT, pedunculopontine tegmentum.

In summary, nuclei associated with anxiety, depression, and fear in the context of aberrant REMS is summarized in **Figure 2**.

## Discussion and outlook

This review has synthesized recent understanding of the neural circuitry underlying mammalian REMS and its functional significance in emotional processing. Despite recent progress, several fundamental questions remain unanswered. First, although cortical involvement in REMS regulation is increasingly recognized, whether it exerts influence via subcortical nuclei requires further elucidation. Second, while the brainstem and hypothalamus are well-established as crucial regulators, the nature of their functional interaction and concurrent coordination in modulating REMS, as well as the identity of genetically defined neuronal subpopulations within these regions need clearer delineation. Third, the activity changes observed from neural recordings during REMS do not inherently establish a causal role in controlling this state. Although optogenetic or chemogenetic manipulations can induce REMS, such artificial activation may introduce non-specific physiological confounds, complicating interpretation. Finally, it is likely that distinct neural subcircuits govern discrete components of REMS, such as its duration, muscle atonia, and ocular movements. We propose that future studies will assess REMS alterations across multiple dimensions, including but not limited to latency, fragmentation, theta power, atonia, and emotion-related behavioral changes.

Functionally, REMS critically modulates emotional processing, yet fundamental questions persist: (1) differential neural mechanisms by which REMS regulates distinct emotions, (2) potential crosstalk between emotional domains mediated by shared REMS pathways, (3) specific functional contribution of REMS-characteristic theta oscillations to affective regulation, (4) contradictory findings such as opposing effects of REMSD on depression and fear memory consolidation. Despite these uncertainties, accumulating evidence positions REMS disruption as a core element in the pathophysiology of negative affect, highlighting REMS-focused interventions, including a circuit-specific neuromodulation or brain rhythm entrainment, as promising therapeutic strategies. Any clinical translation, such as the use of established techniques like deep brain stimulation, must be guided by ethical oversight, social safety considerations, and fully informed consent. Future research must prioritize elucidating emotion-specific REMS circuitry, decoding theta dynamics via multimodal imaging/computational modeling, developing REMS biomarkers for clinical personalization, and translating circuit mechanisms into precise neuromodulation protocols for neuropsychiatric disorders.

Translating circuit mechanisms from basic research into clinical practice will involve several other challenges, in addition to the directions mentioned above. First, the anatomical homology and functional conservation of neural circuits of REMS regulation between rodents and humans require further validation. Second, while techniques like optogenetics allow precise manipulation in animals, achieving comparable precision and safety in humans remains a significant challenge. The development of closed-loop neuromodulation systems for dynamic REMS regulation and the assessment of neurotransmitter-based therapies may represent promising avenues. Future pharmacological strategies could aim to fine-tune neurotransmitter release or develop structural analogs to normalize REMS architecture, thereby alleviating associated emotional symptoms.

## References

[1] Aserinsky E Kleitman N . Regularly occurring periods of eye motility, and concomitant phenomena, during sleep. Sci (New York N.Y.). (1953) 118:273–4. doi: 10.1126/science.118.3062.273, PMID: 13089671

[2] Blumberg MS Lesku JA Libourel PA Schmidt MH Rattenborg NC . What is REM sleep? Curr biol: CB. (2020) 30:R38–r49. doi: 10.1016/j.cub.2019.11.045, PMID: 31910377 PMC6986372

[3] Crick F Mitchison G . The function of dream sleep. Nature. (1983) 304:111–4. doi: 10.1038/304111a0, PMID: 6866101

[4] Luppi PH . Obituary: Michel Jouvet (1925-2017), The father of paradoxical sleep. J Sleep Res. (2017) 26:832–4. doi: 10.1111/jsr.12646, PMID: 29178163

[5] Luppi P-H Chancel A Malcey J Cabrera S Fort P Maciel RM . and Affiliations, Which structure generates paradoxical (REM) sleep: The brainstem, the hypothalamus, the amygdala or the cortex? Sleep Med Rev. (2024) 74:101907. doi: 10.1016/j.smrv.2024.101907, PMID: 38422648

[6] Park SH Weber F . Neural and homeostatic regulation of REM sleep. Front Psychol. (2020) 10.3389/fpsyg.2020.01662 PMC7385183 32793050

[7] Wang Z Fei X Liu X Wang Y Hu Y Peng W . REM sleep is associated with distinct global cortical dynamics and controlled by occipital cortex. Nat Commun. (2022) 13:6896. doi: 10.1038/s41467-022-34720-9, PMID: 36371399 PMC9653484

[8] Sastre JP Buda C Lin JS Jouvet M . Differential c-fos expression in the rhinencephalon and striatum after enhanced sleep-wake states in the cat. Eur J Neurosci. (2000) 12:1397–410. doi: 10.1046/j.1460-9568.2000.00006.x, PMID: 10762368

[9] Koike BDV Farias KS Billwiller F Almeida-Filho D Libourel PA Tiran-Cappello A . Electrophysiological evidence that the retrosplenial cortex displays a strong and specific activation phased with hippocampal theta during paradoxical (REM) sleep. J neurosci: Off J Soc Neurosci. (2017) 37:8003–13. doi: 10.1523/JNEUROSCI.0026-17.2017, PMID: 28729438 PMC6596907

[10] Miyawaki H Watson BO Diba K . Neuronal firing rates diverge during REM and homogenize during non-REM. Sci Rep. (2019) 9:689. doi: 10.1038/s41598-018-36710-8, PMID: 30679509 PMC6345798

[11] Maciel R Yamazaki R Wang D De Laet A Cabrera S Agnorelli C . Is REM sleep a paradoxical state?: Different neurons are activated in the cingulate cortices and the claustrum during wakefulness and paradoxical sleep hypersomnia. Biochem Pharmacol. (2021) 191:114514. doi: 10.1016/j.bcp.2021.114514, PMID: 33713640

[12] Dong Y Li J Zhou M Du Y Liu D . Cortical regulation of two-stage rapid eye movement sleep. Nat Neurosci. (2022) 25:1675–82. doi: 10.1038/s41593-022-01195-2, PMID: 36396977

[13] BrankaČK J ScheffzÜK C Kukushka VI Vyssotski AL Tort ABL Draguhn A . Distinct features of fast oscillations in phasic and tonic rapid eye movement sleep. J Sleep Res. (2012) 21:630–3. doi: 10.1111/j.1365-2869.2012.01037.x, PMID: 22812730

[14] Sánchez-López A Escudero M . Tonic and phasic components of eye movements during REM sleep in the rat. Eur J Neurosci. (2011) 33:2129–38. doi: 10.1111/j.1460-9568.2011.07702.x, PMID: 21645106

[15] Moruzzi G . Active Processes in the brainstem during sleep. Harvey Lect. (1963) 58:233–97. 14272578

[16] Simor P van der Wijk G Nobili L Peigneux P . The microstructure of REM sleep: Why phasic and tonic? Sleep Med Rev. (2020) 52:101305. doi: 10.1016/j.smrv.2020.101305, PMID: 32259697

[17] Bueno-Junior LS Ruckstuhl MS Lim MM Watson BO . The temporal structure of REM sleep shows minute-scale fluctuations across brain and body in mice and humans. Proc Natl Acad Sci United States America. (2023) 120:e2213438120. doi: 10.1073/pnas.2213438120, PMID: 37094161 PMC10161068

[18] Hammer M Jung F Brankačk J Yanovsky Y Tort ABL Draguhn A . Respiration and rapid eye movement (REM) sleep substructure: short versus long episodes. J Sleep Res. (2023) 32:e13777. doi: 10.1111/jsr.13777, PMID: 36398708

[19] Manni R Terzaghi M Glorioso M . Motor-behavioral episodes in REM sleep behavior disorder and phasic events during REM sleep. Sleep. (2009) 32:241–5. doi: 10.1093/sleep/32.2.241, PMID: 19238811 PMC2635588

[20] Sunwoo JS Cha KS Byun JI Kim TJ Jun JS Lim JA . Abnormal activation of motor cortical network during phasic REM sleep in idiopathic REM sleep behavior disorder. Sleep. (2019) 42:zsy227. doi: 10.1093/sleep/zsy227, PMID: 30445515

[21] Campana C Zubler F Gibbs S de Carli F Proserpio P Rubino A . Suppression of interictal spikes during phasic rapid eye movement sleep: a quantitative stereo-electroencephalography study. J Sleep Res. (2017) 26:606–13. doi: 10.1111/jsr.12533, PMID: 28401614

[22] Frauscher B von Ellenrieder N Dubeau F Gotman J . EEG desynchronization during phasic REM sleep suppresses interictal epileptic activity in humans. Epilepsia. (2016) 57:879–88. doi: 10.1111/epi.13389, PMID: 27112123 PMC4949560

[23] Hong J Lozano DE Beier KT Chung S Weber F . Prefrontal cortical regulation of REM sleep. Nat Neurosci. (2023) 26:1820–32. doi: 10.1038/s41593-023-01398-1, PMID: 37735498

[24] Jego S Glasgow SD Herrera CG Ekstrand M Reed SJ Boyce R . Optogenetic identification of a rapid eye movement sleep modulatory circuit in the hypothalamus. Nat Neurosci. (2013) 16:1637–43. doi: 10.1038/nn.3522, PMID: 24056699 PMC4974078

[25] Sapin E Bérod A Léger L Herman PA Luppi PH Peyron C . A very large number of GABAergic neurons are activated in the tuberal hypothalamus during paradoxical (REM) sleep hypersomnia. PloS One. (2010) 5:e11766. doi: 10.1371/journal.pone.0011766, PMID: 20668680 PMC2909908

[26] Renouard L Billwiller F Ogawa K Clément O Camargo N Abdelkarim M . The supramammillary nucleus and the claustrum activate the cortex during REM sleep. Sci Adv. (2015) 1:e1400177. doi: 10.1126/sciadv.1400177, PMID: 26601158 PMC4640625

[27] Wang HL Morales M . Pedunculopontine and laterodorsal tegmental nuclei contain distinct populations of cholinergic, glutamatergic and GABAergic neurons in the rat. Eur J Neurosci. (2009) 29:340–58. doi: 10.1111/j.1460-9568.2008.06576.x, PMID: 19200238 PMC3833361

[28] Martinez-Gonzalez C Wang HL Micklem BR Bolam JP Mena-Segovia J . Subpopulations of cholinergic, GABAergic and glutamatergic neurons in the pedunculopontine nucleus contain calcium-binding proteins and are heterogeneously distributed. Eur J Neurosci. (2012) 35:723–34. doi: 10.1111/j.1460-9568.2012.08002.x, PMID: 22356461

[29] Kroeger D Ferrari LL Petit G Mahoney CE Fuller PM Arrigoni E . Cholinergic, glutamatergic, and GABAergic neurons of the pedunculopontine tegmental nucleus have distinct effects on sleep/wake behavior in mice. J neurosci: Off J Soc Neurosci. (2017) 37:1352–66. doi: 10.1523/JNEUROSCI.1405-16.2016, PMID: 28039375 PMC5296799

[30] Van Dort CJ Zachs DP Kenny JD Zheng S Goldblum RR Gelwan NA . Optogenetic activation of cholinergic neurons in the PPT or LDT induces REM sleep. Proc Natl Acad Sci United States America. (2015) 112:584–9. doi: 10.1073/pnas.1423136112, PMID: 25548191 PMC4299243

[31] Cox J Pinto L Dan Y . Calcium imaging of sleep-wake related neuronal activity in the dorsal pons. Nat Commun. (2016) 7:10763. doi: 10.1038/ncomms10763, PMID: 26911837 PMC4773416

[32] Kumar Yadav R Mallick BN . Dopaminergic- and cholinergic-inputs from substantia nigra and pedunculo-pontine tegmentum, respectively, converge in amygdala to modulate rapid eye movement sleep in rats. Neuropharmacology. (2021) 193:108607. doi: 10.1016/j.neuropharm.2021.108607, PMID: 34023337

[33] Lu J Sherman D Devor M Saper CB . A putative flip-flop switch for control of REM sleep. Nature. (2006) 441:589–94. doi: 10.1038/nature04767, PMID: 16688184

[34] Uchida S Soya S Saito YC Hirano A Koga K Tsuda M . A discrete glycinergic neuronal population in the ventromedial medulla that induces muscle atonia during REM sleep and cataplexy in mice. J neurosci: Off J Soc Neurosci. (2021) 41:1582–96. doi: 10.1523/JNEUROSCI.0688-20.2020, PMID: 33372061 PMC7896014

[35] Clément O Sapin E Bérod A Fort P Luppi PH . Evidence that neurons of the sublaterodorsal tegmental nucleus triggering paradoxical (REM) sleep are glutamatergic. Sleep. (2011) 34:419–23. doi: 10.1093/sleep/34.4.419, PMID: 21461384 PMC3064553

[36] Kubin L . Carbachol models of REM sleep: recent developments and new directions. Arch italiennes biologie. (2001) 139:147–68., PMID: 11256182

[37] Feng H Wen SY Qiao QC Pang YJ Wang SY Li HY . Orexin signaling modulates synchronized excitation in the sublaterodorsal tegmental nucleus to stabilize REM sleep. Nat Commun. (2020) 11:3661. doi: 10.1038/s41467-020-17401-3, PMID: 32694504 PMC7374574

[38] Kashiwagi M Beck G Kanuka M Arai Y Tanaka K Tatsuzawa C . A pontine-medullary loop crucial for REM sleep and its deficit in Parkinson’s disease. Cell. (2024) 187:6272–89. doi: 10.1016/j.cell.2024.08.046, PMID: 39303715

[39] Valencia Garcia S Brischoux F Clément O Libourel PA Arthaud S Lazarus M . Ventromedial medulla inhibitory neuron inactivation induces REM sleep without atonia and REM sleep behavior disorder. Nat Commun. (2018) 9:504. doi: 10.1038/s41467-017-02761-0, PMID: 29402935 PMC5799338

[40] Morales FR Sampogna S Rampon C Luppi PH Chase MH . Brainstem glycinergic neurons and their activation during active (rapid eye movement) sleep in the cat. Neuroscience. (2006) 142:37–47. doi: 10.1016/j.neuroscience.2006.05.066, PMID: 16891059

[41] Weber F Chung S Beier KT Xu M Luo L Dan Y . Control of REM sleep by ventral medulla GABAergic neurons. Nature. (2015) 526:435–8. doi: 10.1038/nature14979, PMID: 26444238 PMC4852286

[42] Stucynski JA Schott AL Baik J Chung S Weber F . Regulation of REM sleep by inhibitory neurons in the dorsomedial medulla. Curr biol: CB. (2022) 32:37–50.e6. doi: 10.1016/j.cub.2021.10.030, PMID: 34735794 PMC8752505

[43] Gervasoni D Darracq L Fort P Soulière F Chouvet G Luppi PH . Electrophysiological evidence that noradrenergic neurons of the rat locus coeruleus are tonically inhibited by GABA during sleep. Eur J Neurosci. (1998) 10:964–70. doi: 10.1046/j.1460-9568.1998.00106.x, PMID: 9753163

[44] Gervasoni D Peyron C Rampon C Barbagli B Chouvet G Urbain N . Role and origin of the GABAergic innervation of dorsal raphe serotonergic neurons. J Neurosci. (2000) 20:4217–25. doi: 10.1523/JNEUROSCI.20-11-04217.2000, PMID: 10818157 PMC6772634

[45] Goutagny R Luppi PH Salvert D Lapray D Gervasoni D Fort P . Role of the dorsal paragigantocellular reticular nucleus in paradoxical (rapid eye movement) sleep generation: a combined electrophysiological and anatomical study in the rat. Neuroscience. (2008) 152:849–57. doi: 10.1016/j.neuroscience.2007.12.014, PMID: 18308473

[46] Sirieix C Gervasoni D Luppi PH Léger L . Role of the lateral paragigantocellular nucleus in the network of paradoxical (REM) sleep: an electrophysiological and anatomical study in the rat. PloS One. (2012) 7:e28724. doi: 10.1371/journal.pone.0028724, PMID: 22235249 PMC3250413

[47] Yang SR Hu ZZ Luo YJ Zhao YN Sun HX Yin D . The rostromedial tegmental nucleus is essential for non-rapid eye movement sleep. PloS Biol. (2018) 16:e2002909. doi: 10.1371/journal.pbio.2002909, PMID: 29652889 PMC5919677

[48] Zhao YN Jiang JB Tao SY Zhang Y Chen ZK Qu WM . GABAergic neurons in the rostromedial tegmental nucleus are essential for rapid eye movement sleep suppression. Nat Commun. (2022) 13:7552. doi: 10.1038/s41467-022-35299-x, PMID: 36477665 PMC9729601

[49] Weber F Hoang Do JP Chung S Beier KT Bikov M Saffari Doost M . Regulation of REM and non-REM sleep by periaqueductal GABAergic neurons. Nat Commun. (2018) 9:354. doi: 10.1038/s41467-017-02765-w, PMID: 29367602 PMC5783937

[50] Sulaman BA Wang S Tyan J Eban-Rothschild A . Neuro-orchestration of sleep and wakefulness. Nat Neurosci. (2023) 26:196–212. doi: 10.1038/s41593-022-01236-w, PMID: 36581730 PMC12714371

[51] Chen ZK Dong H Liu CW Liu WY Zhao YN Xu W . A cluster of mesopontine GABAergic neurons suppresses REM sleep and curbs cataplexy. Cell Discov. (2022) 8:115. doi: 10.1038/s41421-022-00456-5, PMID: 36280664 PMC9592589

[52] Clément O Sapin E Libourel PA Arthaud S Brischoux F Fort P . The lateral hypothalamic area controls paradoxical (REM) sleep by means of descending projections to brainstem GABAergic neurons. J neurosci: Off J Soc Neurosci. (2012) 32:16763–74. doi: 10.1523/JNEUROSCI.1885-12.2012, PMID: 23175830 PMC6621764

[53] Feng H Qiao QC Luo QF Zhou JY Lei F Chen Y . Orexin neurons to sublaterodorsal tegmental nucleus pathway prevents sleep onset REM sleep-like behavior by relieving the REM sleep pressure. Research-China. (2024) 7:0355. doi: 10.34133/research.0355, PMID: 38694202 PMC11062508

[54] Chung S Weber F Zhong P Tan CL Nguyen TN Beier KT . Identification of preoptic sleep neurons using retrograde labelling and gene profiling. Nature. (2017) 545:477–81. doi: 10.1038/nature22350, PMID: 28514446 PMC5554302

[55] Miracca G Anuncibay-Soto B Tossell K Yustos R Vyssotski AL Franks NP . NMDA receptors in the lateral preoptic hypothalamus are essential for sustaining NREM and REM sleep. J neurosci: Off J Soc Neurosci. (2022) 42:5389–409. doi: 10.1523/JNEUROSCI.0350-21.2022, PMID: 35649726 PMC7613025

[56] Chen KS Xu M Zhang Z Chang WC Gaj T Schaffer DV . and non-REM sleep. Neuron. (2018) 97:1168–1176.e4. doi: 10.1016/j.neuron.2018.02.005, PMID: 29478915

[57] Billwiller F Castillo L Elseedy H Ivanov AI Scapula J Ghestem A . GABA-glutamate supramammillary neurons control theta and gamma oscillations in the dentate gyrus during paradoxical (REM) sleep. Brain struct Funct. (2020) 225:2643–68. doi: 10.1007/s00429-020-02146-y, PMID: 32970253 PMC7674372

[58] Soussi R Zhang N Tahtakran S Houser CR Esclapez M . Heterogeneity of the supramammillary-hippocampal pathways: evidence for a unique GABAergic neurotransmitter phenotype and regional differences. Eur J Neurosci. (2010) 32:771–85. doi: 10.1111/j.1460-9568.2010.07329.x, PMID: 20722723 PMC2974797

[59] Liu K Kim J Kim DW Zhang YS Bao H Denaxa M . Lhx6-positive GABA-releasing neurons of the zona incerta promote sleep. Nature. (2017) 548:582–7. doi: 10.1038/nature23663, PMID: 28847002 PMC5958617

[60] Vidal-Ortiz A Blanco-Centurion C Shiromani PJ . Unilateral optogenetic stimulation of Lhx6 neurons in the zona incerta increases REM sleep. Sleep. (2024) 47:zsad217. doi: 10.1093/sleep/zsad217, PMID: 37599437 PMC11502959

[61] Chancel A Fort P Luppi PH . The role of the hypothalamic Lhx6 GABAergic neurons in REM sleep control. Sleep. (2024) 47. doi: 10.1093/sleep/zsad331, PMID: 38159085 PMC10925945

[62] Wang YQ Li R Wang DR Cherasse Y Zhang Z Zhang MQ . Adenosine A(2A) receptors in the olfactory bulb suppress rapid eye movement sleep in rodents. Brain struct Funct. (2017) 222:1351–66. doi: 10.1007/s00429-016-1281-2, PMID: 27485749

[63] Puskar P Sengupta T Sharma B Nath SS Mallick H Akhtar N . Changes in sleep-wake cycle after microinjection of agonist and antagonist of endocannabinoid receptors at the medial septum of rats. Physiol Behav. (2021) 237:113448. doi: 10.1016/j.physbeh.2021.113448, PMID: 33957148

[64] Hasegawa E Miyasaka A Sakurai K Cherasse Y Li Y Sakurai T . Rapid eye movement sleep is initiated by basolateral amygdala dopamine signaling in mice. Sci (New York N.Y.). (2022) 375:994–1000. doi: 10.1126/science.abl6618, PMID: 35239361

[65] Ba W Nollet M Yin C Yu X Wong S Miao A . A REM-active basal ganglia circuit that regulates anxiety. Curr biol: CB. (2024) 34:3301–3314.e4. doi: 10.1016/j.cub.2024.06.010, PMID: 38944034

[66] Zhang ZH Zhang W Fang YY Wang N Liu GY Zou N . A potentiation of REM sleep-active neurons in the lateral habenula may be responsible for the sleep disturbance in depression. Curr Biol. (2024) 34:3287–300. doi: 10.1016/j.cub.2024.05.075, PMID: 38944036

[67] Marriott BA Do AD Portet C Thellier F Goutagny R Jackson J . Brain-state-dependent constraints on claustrocortical communication and function. Cell Rep. (2024) 43:113620. doi: 10.1016/j.celrep.2023.113620, PMID: 38159273

[68] Wunderlin M Studler M Gianotti LRR Züst MA Knoch D . Interindividual differences in mindfulness are linked to sleep-EEG characteristics. Sleep. (2024) 47:zsae101. doi: 10.1093/sleep/zsae101, PMID: 38676404 PMC11236951

[69] Reichardt R Király A Szőllősi Á Racsmány M Simor P . A daytime nap with REM sleep is linked to enhanced generalization of emotional stimuli. J Sleep Res. (2024) 33:e14177. doi: 10.1111/jsr.14177, PMID: 38369938

[70] Pronier É Morici JF Girardeau G . The role of the hippocampus in the consolidation of emotional memories during sleep. Trends Neurosci. (2023) 46:912–25. doi: 10.1016/j.tins.2023.08.003, PMID: 37714808

[71] Pesonen AK Koskinen MK Vuorenhela N Halonen R Mäkituuri S Selin M . The effect of REM-sleep disruption on affective processing: A systematic review of human and animal experimental studies. Neurosci Biobehav Rev. (2024) 162:105714. doi: 10.1016/j.neubiorev.2024.105714, PMID: 38729279

[72] Aime M . To “feel” better, sleep on it! Sci (New York N.Y.). (2023) 382:528. doi: 10.1126/science.adk3894, PMID: 37917675

[73] Yin M Chen Y Zheng H Pu T Marshall C Wu T . Assessment of mouse cognitive and anxiety-like behaviors and hippocampal inflammation following a repeated and intermittent paradoxical sleep deprivation procedure. Behav Brain Res. (2017) 321:69–78. doi: 10.1016/j.bbr.2016.12.034, PMID: 28043900

[74] da Silva Rocha-Lopes J MaChado RB Suchecki D . Chronic REM sleep restriction in juvenile male rats induces anxiety-like behavior and alters monoamine systems in the amygdala and hippocampus. Mol Neurobiol. (2018) 55:2884–96. doi: 10.1007/s12035-017-0541-3, PMID: 28455701

[75] Liu H Huang X Li Y Xi K Han Y Mao H . TNF signaling pathway-mediated microglial activation in the PFC underlies acute paradoxical sleep deprivation-induced anxiety-like behaviors in mice. Brain Behav Immun. (2022) 100:254–66. doi: 10.1016/j.bbi.2021.12.006, PMID: 34915154

[76] Fan FF Vetrivelan R Yang Y Guo ZN Lu J . Role of pontine sub-laterodorsal tegmental nucleus (SLD) in rapid eye movement (REM) sleep, cataplexy, and emotion. CNS Neurosci Ther. (2023) 29:1192–6. doi: 10.1111/cns.14074, PMID: 36585816 PMC10018081

[77] Palagini L Baglioni C Ciapparelli A Gemignani A Riemann D . REM sleep dysregulation in depression: state of the art. Sleep Med Rev. (2013) 17:377–90. doi: 10.1016/j.smrv.2012.11.001, PMID: 23391633

[78] Wang YQ Li R Zhang MQ Zhang Z Qu WM Huang ZL . The neurobiological mechanisms and treatments of REM sleep disturbances in depression. Curr Neuropharmacol. (2015) 13:543–53. doi: 10.2174/1570159X13666150310002540, PMID: 26412074 PMC4790401

[79] Omichi C Kadotani H Sumi Y Ubara A Nishikawa K Matsuda A . Prolonged sleep latency and reduced REM latency are associated with depressive symptoms in a Japanese working population. Int J Environ Res Public Health. (2022) 19:2112. doi: 10.3390/ijerph19042112, PMID: 35206296 PMC8872621

[80] Huang M Bliwise DL Hall MH Johnson DA Sloan RP Shah A . Association of depressive symptoms with sleep disturbance: A co-twin control study. Ann Behav Med. (2022) 56:245–56. doi: 10.1093/abm/kaab040, PMID: 33991086 PMC8887572

[81] Zhao Y Zhang H Zhang Y Fang Z Xu C . Rapid eye movement sleep deprivation enhances adenosine receptor activation and the CREB1/YAP1/c-myc axis to alleviate depressive-like behaviors in rats. ACS Chem Neurosci. (2022) 13:2298–308. doi: 10.1021/acschemneuro.2c00167, PMID: 35838172

[82] Ma HY Xu YF Qiao D Wen YJ Zhao T Wang XP . Abnormal sleep features in adolescent MDD and its potential in diagnosis and prediction of early efficacy. Sleep Med. (2023) 106:116–22. doi: 10.1016/j.sleep.2023.01.021, PMID: 36740544

[83] Zhang C Zhu DM Zhang Y Chen T Liu S Chen J . Neural substrates underlying REM sleep duration in patients with major depressive disorder: A longitudinal study combining multimodal MRI data. J Affect Disord. (2024) 344:546–53. doi: 10.1016/j.jad.2023.10.090, PMID: 37848093

[84] Rahmani M Rahmani F Rezaei N . The brain-derived neurotrophic factor: missing link between sleep deprivation, insomnia, and depression. Neurochem Res. (2020) 45:221–31. doi: 10.1007/s11064-019-02914-1, PMID: 31782101

[85] Saadati N Bananej M Khakpai F Zarrindast MR Alibeik H . Synergistic antidepressant effects of citalopram and SB-334867 in the REM sleep-deprived mice: Possible role of BDNF. Pharmacol Biochem Behav. (2022) 219:173449. doi: 10.1016/j.pbb.2022.173449, PMID: 35973584

[86] Zhu Y Wu T Jiao Q Chai H Wang Y Tian C . Acute REM sleep deprivation alleviated depression-like behavior mediated by inhibiting VIP neurons in the mPFC. Sci Adv. (2025) 11:eadx2666. doi: 10.1126/sciadv.adx2666, PMID: 40929273 PMC12422183

[87] Katzman MA Katzman MP . Neurobiology of the orexin system and its potential role in the regulation of hedonic tone. Brain Sci. (2022) 12:150. doi: 10.3390/brainsci12020150, PMID: 35203914 PMC8870430

[88] Stanquini LA Sartim AG Joca SRL . Orexin A injection into the ventral medial prefrontal cortex induces antidepressant-like effects: Possible involvement of local Orexin-1 and Trk receptors. Behav Brain Res. (2020) 395:112866. doi: 10.1016/j.bbr.2020.112866, PMID: 32827568

[89] Leitner C Dalle Piagge F Tomic T Nozza F Fasiello E Castronovo V . Sleep alterations in major depressive disorder and insomnia disorder: A network meta-analysis of polysomnographic studies. Sleep Med Rev. (2025) 80:102048. doi: 10.1016/j.smrv.2025.102048, PMID: 40054014

[90] Riemann D Krone LB Wulff K Nissen C . Sleep, insomnia, and depression. Neuropsychopharmacol. (2020) 45:74–89. doi: 10.1038/s41386-019-0411-y, PMID: 31071719 PMC6879516

[91] Modell S Ising M Holsboer F Lauer CJ . The Munich vulnerability study on affective disorders: premorbid polysomnographic profile of affected high-risk probands. Biol Psychiatry. (2005) 58:694–9. doi: 10.1016/j.biopsych.2005.05.004, PMID: 16018976

[92] Kheirkhah M Duncan WC Yuan Q Wang PR Jamalabadi H Leistritz L . REM density predicts rapid antidepressant response to ketamine in individuals with treatment-resistant depression. Neuropsychopharmacol. (2025) 50:941–6. doi: 10.1038/s41386-025-02066-7, PMID: 39955416 PMC12032024

[93] Boafo A Armitage R Greenham S Tavakoli P Dale A Nixon A . Sleep architecture in adolescents hospitalized during a suicidal crisis. Sleep Med. (2019) 56:41–6. doi: 10.1016/j.sleep.2018.12.018, PMID: 30737143

[94] Lechinger J Koch J Weinhold SL Seeck-Hirschner M Stingele K Kropp-Näf C . REM density is associated with treatment response in major depression: Antidepressant pharmacotherapy vs. psychotherapy. J Psychiatr Res. (2021) 133:67–72. doi: 10.1016/j.jpsychires.2020.12.009, PMID: 33310502

[95] Liu S Chen J Guan L Xu L Cai H Wang J . The brain, rapid eye movement sleep, and major depressive disorder: A multimodal neuroimaging study. Prog Neuropsychopharmacol Biol Psychiatry. (2025) 136:111151. doi: 10.1016/j.pnpbp.2024.111151, PMID: 39326695

[96] De la Fuente JM Bobes J Vizuete C Mendlewicz J . Sleep-EEG in borderline patients without concomitant major depression: a comparison with major depressives and normal control subjects. Psychiatry Res. (2001) 105:87–95. doi: 10.1016/S0165-1781(01)00330-4, PMID: 11740978

[97] de Lange SC Tissink E Bresser T Savage JE Posthuma D Heuvel MPvd . Multimodal brain imaging of insomnia, depression and anxiety symptoms indicates transdiagnostic commonalities and differences. Nat Ment Health. (2025) 3:517–29. doi: 10.1038/s44220-025-00412-8

[98] Myers KM Davis M . Mechanisms of fear extinction. Mol Psychiatry. (2007) 12:120–50. doi: 10.1038/sj.mp.4001939, PMID: 17160066

[99] Pace-Schott EF Germain A Milad MR . Effects of sleep on memory for conditioned fear and fear extinction. Psychol Bull. (2015) 141:835–57. doi: 10.1037/bul0000014, PMID: 25894546 PMC4486610

[100] Gazea M Rio-Bermudez CD Nissen C Adamantidis AR . Chapter 17 - functions and circuits of REM sleep. In: Handbook of Behavioral Neuroscience. Dringenberg HC: Elsevier, (2019). 249–67.

[101] Pitman RK Rasmusson AM Koenen KC Shin LM Orr SP Gilbertson MW . Biological studies of post-traumatic stress disorder. Nat Rev Neurosci. (2012) 13:769–87. doi: 10.1038/nrn3339, PMID: 23047775 PMC4951157

[102] Merians AN Spiller T Harpaz-Rotem I Krystal JH Pietrzak RH . Post-traumatic stress disorder. Med Clin North Am. (2023) 107:85–99. doi: 10.1016/j.mcna.2022.04.003, PMID: 36402502

[103] Maercker A Cloitre M Bachem R Schlumpf YR Khoury B Hitchcock C . Complex post-traumatic stress disorder. Lancet. (2022) 400:60–72. doi: 10.1016/S0140-6736(22)00821-2, PMID: 35780794

[104] Ressler KJ Berretta S Bolshakov VY Rosso IM Meloni EG Rauch SL . Post-traumatic stress disorder: clinical and translational neuroscience from cells to circuits. Nat Rev Neurol. (2022) 18:273–88. doi: 10.1038/s41582-022-00635-8, PMID: 35352034 PMC9682920

[105] Kobayashi I Boarts JM Delahanty DL . Polysomnographically measured sleep abnormalities in PTSD: a meta-analytic review. Psychophysiology. (2007) 44:660–9. doi: 10.1111/j.1469-8986.2007.537.x, PMID: 17521374

[106] Ansbjerg MB Sandahl H Baandrup L Jennum P Carlsson J . Sleep impairments in refugees diagnosed with post-traumatic stress disorder: a polysomnographic and self-report study. Eur J Psychotraumatol. (2023) 14:2185943. doi: 10.1080/20008066.2023.2185943, PMID: 36971225 PMC10044313

[107] Bottari SA Trifilio ER Rohl B Wu SS Miller-Sellers D Waldorff I . Optimizing transcutaneous vagus nerve stimulation parameters for sleep and autonomic function in veterans with posttraumatic stress disorder with or without mild traumatic brain injury. Sleep. (2025) 48:zsaf152. doi: 10.1093/sleep/zsaf152, PMID: 40489292

[108] Tseng YT Zhao B Chen S Ye J Liu J Liang L . The subthalamic corticotropin-releasing hormone neurons mediate adaptive REM-sleep responses to threat. Neuron. (2022) 110:1223–1239.e8. doi: 10.1016/j.neuron.2021.12.033, PMID: 35065715

[109] Boyce R Glasgow SD Williams S Adamantidis A . Causal evidence for the role of REM sleep theta rhythm in contextual memory consolidation. Sci (New York N.Y.). (2016) 352:812–6. doi: 10.1126/science.aad5252, PMID: 27174984

[110] Wen YJ Yang WJ Guo CN Qiu MH Kroeger D Niu JG . Pontine control of rapid eye movement sleep and fear memory. CNS Neurosci Ther. (2023) 29:1602–14. doi: 10.1111/cns.14123, PMID: 36794544 PMC10173714

[111] Hong J Choi K Fuccillo MV Chung S Weber F . Infralimbic activity during REM sleep facilitates fear extinction memory. Curr biol: CB. (2024) 34:2247–2255.e5. doi: 10.1016/j.cub.2024.04.018, PMID: 38714199 PMC11111341

[112] Menz MM Rihm JS Salari N Born J Kalisch R Pape HC . The role of sleep and sleep deprivation in consolidating fear memories. Neuroimage. (2013) 75:87–96. doi: 10.1016/j.neuroimage.2013.03.001, PMID: 23501052

[113] Louie K Wilson MA . Temporally structured replay of awake hippocampal ensemble activity during rapid eye movement sleep. Neuron. (2001) 29:145–56. doi: 10.1016/S0896-6273(01)00186-6, PMID: 11182087

[114] Tadros T Krishnan GP Ramyaa R Bazhenov M . Sleep-like unsupervised replay reduces catastrophic forgetting in artificial neural networks. Nat Commun. (2022) 13:7742. doi: 10.1038/s41467-022-34938-7, PMID: 36522325 PMC9755223

[115] Rihm JS Rasch B . Replay of conditioned stimuli during late REM and stage N2 sleep influences affective tone rather than emotional memory strength. Neurobiol Learn Mem. (2015) 122:142–51. doi: 10.1016/j.nlm.2015.04.008, PMID: 25933506

[116] Seo J Pace-Schott EF Milad MR Song H Germain A . Partial and total sleep deprivation interferes with neural correlates of consolidation of fear extinction memory. Biol Psychiatry Cognit Neurosci Neuroimaging. (2021) 6:299–309. doi: 10.1016/j.bpsc.2020.09.013, PMID: 33279459

[117] Lerner I Lupkin SM Tsai A Khawaja A Gluck MA . Sleep to remember, sleep to forget: Rapid eye movement sleep can have inverse effects on recall and generalization of fear memories. Neurobiol Learn Mem. (2021) 180:107413. doi: 10.1016/j.nlm.2021.107413, PMID: 33609741

